# Modelling nurses’ use of local anaesthesia for intravenous cannulation and arterial blood gas sampling: A cross-sectional study

**DOI:** 10.1016/j.heliyon.2020.e03428

**Published:** 2020-03-03

**Authors:** Fatimah Yahya Alobayli, Ian Blackman

**Affiliations:** a King Saud Medical City, Ulaishah, Riyadh, 12746, Saudi Arabia; b Flinders University, School of Nursing & Midwifery, Sturt East Buildings, Bedford Park, SA, 5042, Australia

**Keywords:** Pharmacology, Critical care, Evidence-based medicine, Clinical research, Nursing, Needle insertion, Arterial blood gases, Intravenous cannulation, Local anaesthesia, Modelling, Nurses, Pain reduction

## Abstract

**Background:**

Needle insertions are painful, yet they are frequently performed for adults and children without using local anaesthetic (LA) to minimise pain and anxiety.

**Objectives:**

A hypothetical model was formulated to explore the factors related to Saudi nurses’ self-reported readiness to use LA prior to undertaking parenteral procedures in their workplaces.

**Design:**

This was an exploratory, cross-sectional study.

**Methods:**

Four hundred seventy-five nurses were recruited from one hospital in Saudi Arabia. We considered eighteen latent variables related to nurses’ attitudes and ability to pursue six roles associated with LA before needle procedures. A model was created to identify the staff attitudes and self-efficacy pathways influencing readiness to use LA.

**Results:**

The nurses' readiness to use LA before needle procedures was directly predicted by organisational factors (e.g., hospital policy, doctors' orders), procedural time constraints, underestimation of needle pain, patient characteristics and medical conditions, nurses’ knowledge and skills related to LA, and parenteral procedure practices.

**Conclusions:**

Nurses' readiness to use LA was influenced by their beliefs about certain aspects of their practice and the nature of patients’ presenting problems.

**Impact statement:**

Identifying factors that affect LA use helps us understand this issue and may assist policymakers in developing nursing practice.

## 1 Introduction

Needle-related procedures, such as intravenous (IV) cannulation and arterial blood gas (ABG) sampling, are painful but frequently required by patients during hospital treatment. Local anaesthetics (LA) are used to relieve pain associated with such procedures in many countries (Hudson et al., 2006; Jimenez et al., 2006). However, nurses in Saudi Arabia routinely perform the procedures without using LA, thus subjecting patients to unnecessary pain and anxiety. Their practices seem against evidence and recommendations regarding the effectiveness of LA for reducing procedural pain (Crowley et al., 2011; McGowan, 2014; Page and Taylor, 2010).

### 1.1 Aims

The study examined the possible factors associated with LA use for needling procedures among Saudi nurses, with a specific focus on the following: hospital organisational structures; patient pain-related and other demographic factors; nurses’ knowledge and skills related to LA and needling procedures.

### 1.2 Background

Pain management for minor procedures, such as IV cannulation and ABG, is low priority in many institutions in Saudi Arabia, and LA is almost never used, even in paediatric settings. Additionally, no studies have been conducted locally to support the use of LA for such procedures. Consequently, patients are often subjected to unnecessary procedural pain, which produces distress and can have negative consequences. Needle phobia affects at least 10% of the population (Yu, 2012), and needle-related procedures often cause considerable pain and anxiety, particularly for patients who must endure multiple attempts due to difficult cannulations (McGowan, 2014). ABG has a high rate of failed attempts because of the artery's anatomical location (Hajiseyedjavady et al., 2012). Procedural pain can also make the practitioner uneasy (Hajiseyedjavady et al., 2012).

Researchers note that alleviating ABG pain is directly related to success of sampling. Additionally, using LA for IV cannulation and ABG sampling can increase the success rates of needle access because patients can remain immobile during the procedure (Hudson et al., 2006). By contrast, failed needle-insertion attempts limit future vascular access and cause patients unnecessary distress and trauma, thereby making subsequent attempts challenging and causing patients symptoms such as anxiety, nausea, increased heart rate, and fainting (Dougherty, 2011). Unmanaged procedural pain can have long-term negative impacts on nervous system development, pain sensitivity, and emotional wellbeing (Yu, 2012), and may cause patients to avoid healthcare follow-ups. This can delay treatment until the late stages of illness, which financially strains the health system, patients, and patients’ families (McGowan, 2014; Yu, 2012).

## 2 Methods

### 2.1 Design

This exploratory cross-sectional study employed survey questionnaires to identify the factors influencing LA use for procedural pain as reported by Saudi nurses. Two instruments using Likert-type response scales were employed: one measured nurses’ attitudes towards factors influencing LA use for IV cannulation and ABG (strongly agree to strongly disagree) and the second measured their ability (i.e. self-efficacy) to use LA and perform needle insertions (very easy to very difficult). Table 1 presents the survey items.

**Table 1:** Description of the factors that influence nurses’ use of local anaesthetic (or not) as used in the survey.

Name and number of the latent variable	Name and number of the manifest variable
Age	Years
Gender	Male = 1, Female = 2
Nationality	Saudi = 1, Filipino = 2, Indian = 3, Other = 4
Highest education	Diploma = 1, College diploma = 2, Bachelor's degree = 3, Master's degree = 4
Current nursing position	Staff nurse = 1, Supervisor/head nurse = 2
Hospital department	Critical settings = 1, Medical = 2, Surgical = 3, Paediatrics = 4, Other = 5
Years of experience	<2 years = 1, 2–5 years = 2, > 5–10 years = 3, >10 years = 4
Experience of needle insertion	IV = 1, IV and ABG = 2, None = 3
Ever used LA	Yes = 1, No = 2
IV/ABG procedures done to self	IV = 1, IV and ABG = 2, None = 3
Ever trained/taught to use LA	Yes = 1, No = 2
**Nurses' attitudes towards LA use** (Scale categories: strongly agree, agree, disagree, strongly disagree, not sure)	**Organisational factors:** Q1 Hospital policies and procedures influence my use of LA Q5 There should be a doctor's order to use LA for these procedures Q11 The cost of medication (LA) limits its availability in hospital Q21 If the policy gives clear direction about LA use, I would follow it
	**Procedural factors:** Q2 The extra time needed is a barrier to my use of LA Q3 It is acceptable to delay the treatment by giving LA Q10 My experience allows me to perform insertion in a less painful way Q12 Topical LA should be used for needle procedures Q15 The size of the cannula determines whether I use LA Q17 LA should be used for ABG procedures Q18 If I could use fast-acting painless intradermal LA (J-tip), I would
	**Pain-related factors:** Q4 There is no point causing additional pain by giving injectable LA Q6 Pain from these procedures is given a low priority by doctors Q7 Patients can tolerate the pain associated with needle insertions Q8 Pain associated with needle punctures is only minor Q9 I think even when LA is used, it will not make a big difference Q19 Less pain and distress improves successful insertion rates
	**Patient-related factors:** Q13 LA should be routinely given to children Q14 Adults can be given the choice to have LA or not for procedures Q16 Patient's medical condition determines whether I use LA or not Q20 Patient's satisfaction is higher when LA is used
**Nurses' ability to use LA** (Scale categories: very easy, easy, difficult, very difficult, not sure)	**Nursing knowledge:** Q22 Understanding key aspects of LA used for cannulation and ABG Q27 Recognising the desired effects of the medication (LA) Q28 Identifying the major side effects of the medication (LA)
	**Nursing skills:** Q23 Administering injectable LA for these procedures Q24 Prioritising my work time to deliver LA for these procedures Q25 Selecting the correct route for administering LA according to the procedure Q26 Determining the patient's level of discomfort/pain during the procedure Q29 Relaxing an anxious child when trying to insert the needle without LA Q30 Inserting the needle successfully even when LA injection swells the skin Q31 Using other strategies, such as ice application or behavioural interventions

LA = local anaesthetic; IV = intravenous; ABG = arterial blood gas.

**Figure 1:**
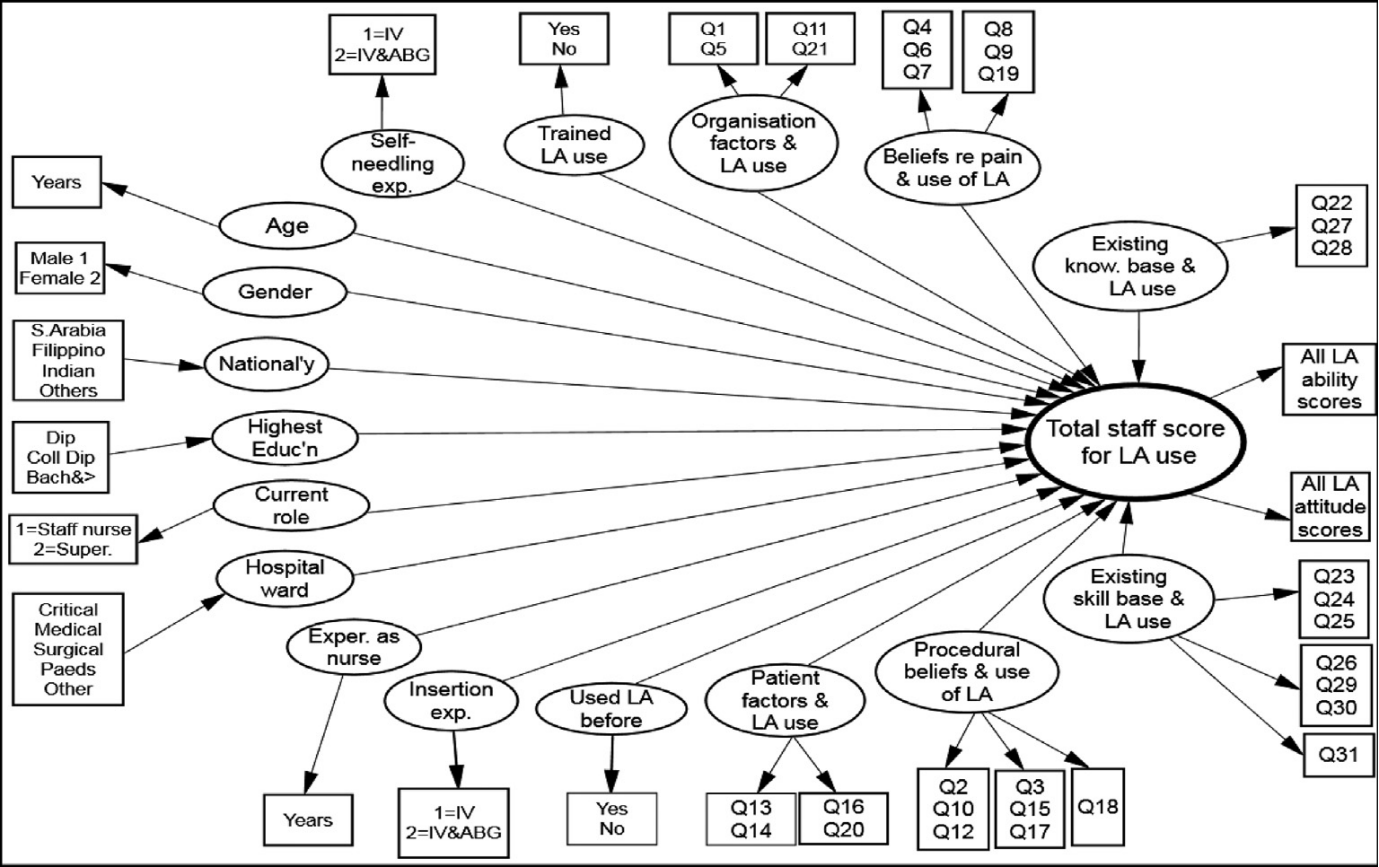
Hypothetical model predicting the variables that influence the use of local anaesthetics for intravenous and arterial blood gas procedures. IV = intravenous; LA = local anaesthetic; Dip = diploma; Coll Dip = college diploma; Bach = Bachelor's degree; Super = supervisor; Paeds = paediatrics; Educ'n = education; Exper = experience; exp. = experience; know. = knowledge; ABG = arterial blood gas.

We hypothesised that all 17 variables (derived from the survey items) would have significant associations with nurses’ LA use (Figure 1). These latent variables (LV) are shown as ellipses in Figure 1, which are reflected by indicators or manifest variables (shown as rectangles) that arise from items completed by the participating nurses.

### 2.2 Sample and participants

A convenience sample of 475 nurses was obtained from a large government hospital in Saudi Arabia. Nurses serving in any nursing role (e.g., staff nurse, supervisor) were included; nursing assistants, interns, and students were excluded. All hospital departments where needling procedures were often carried out were considered.

### 2.3 Data analysis

A path analysis was utilised to explore the factors associated with non-use of LA by Saudi nurses, as well as understand their interactive effects of these factors. We used SmartPLS (version 2.0; Hansmann and Ringle, 2004) to perform structural equation modelling to explain the variance in LA use explained by these abovementioned factors.

### 2.4 Validity and reliability/rigour

#### 2.4.1 Instrument development

The survey questions were generated based on the literature review. Additional items were generated based on the researcher's own experience with IV cannulation and ABG procedures in hospital. A pilot study was conducted on a small group of nurses having experience with needle related procedures to improve the reliability of the instrument, which helped refining the survey questions for clarity and proper phrasing to avoid misunderstandings or misinterpretations of the questions.

#### 2.4.2 Rasch scaling

As we used Likert scales (i.e., ordinal measures) to evaluate nurses' attitudes and ability to use LA, statistics for interval/ratio data were deemed inappropriate (Grimby et al., 2012). For similar reasons, we opted for Rasch analysis to measure reliability, instead of Cronbach's alpha, as the latter cannot confirm if the survey items are unidimensional (Sijtsma, 2009). Rasch analysis measures the unidimensionality of survey items individually by assessing if an item measures the same underlying attributes of the LVs; thus, a strong assumption can be made about each survey item's quality, which is useful for considering whether to eliminate it (Blackman et al., 2015). Thus, all survey items were kept for further analysis as they fit within the acceptable range of the Infit and Outfit means square criteria.

### 2.5 Ethical approval

Ethical approval was given by the Social and Behavioural Research Ethics Committee at Flinders University in Australia and an institutional review board from King Saud Medical City in Saudi Arabia (where the study was conducted). Participants were given the questionnaire along with two letters to obtain informed consent (an introductory letter and an information sheet). The informed consent sheets outlined the study's purpose and the participants' rights (i.e., voluntary participation, confidentiality, and a privacy guarantee), and stated that no harm or discomfort would result from taking part.

## 3 Results

A total of 475 nurses completed the survey (of 600 surveys distributed, response rate 79%). Table 2 shows respondents’ demographic characteristics.

**Table 2:** The demographic data of the study population.

Demographic Data		Frequency *N* = 475	Percent
Age (years)	Under 30	191	40.2
	30–39	202	42.5
	40–49	62	13.1
	50 and above	20	4.2
Gender	Male	16	3.4
	Female	459	96.6
Nationality	Saudi	128	26.9
	Filipino	172	36.2
	Indian	159	33.5
	Others	16	3.4
Highest education	Diploma	173	36.4
	College diploma	65	13.7
	Bachelor's degree and above	237	49.9
Current position	Staff nurse	430	90.5
	Supervisor/head nurse	45	9.5
Hospital departments	Critical settings	100	21.1
	Medical	158	33.3
	Surgical	76	16.0
	Paediatrics	76	16.0
	Others	65	13.7
Working experience	<2 years	66	13.9
	2–5 years	138	29.1
	>5–10 years	145	30.5
	>10 years	126	26.5
Insertion experience	IV cannulation	225	47.4
	IV and ABG	235	49.5
	None	15	3.2
Ever used LA for IV or ABG	Yes	84	17.7
	No	391	82.3
Needle insertion done to you	IV cannulation	297	62.5
	IV and ABG	114	24.0
	None	64	13.5
Taught/trained to use LA	Yes	77	16.2
	No	398	83.8

LA = local anaesthetic; IV = intravenous; ABG = arterial blood gas.

### 3.1 Descriptive analysis

**Figure 2:**
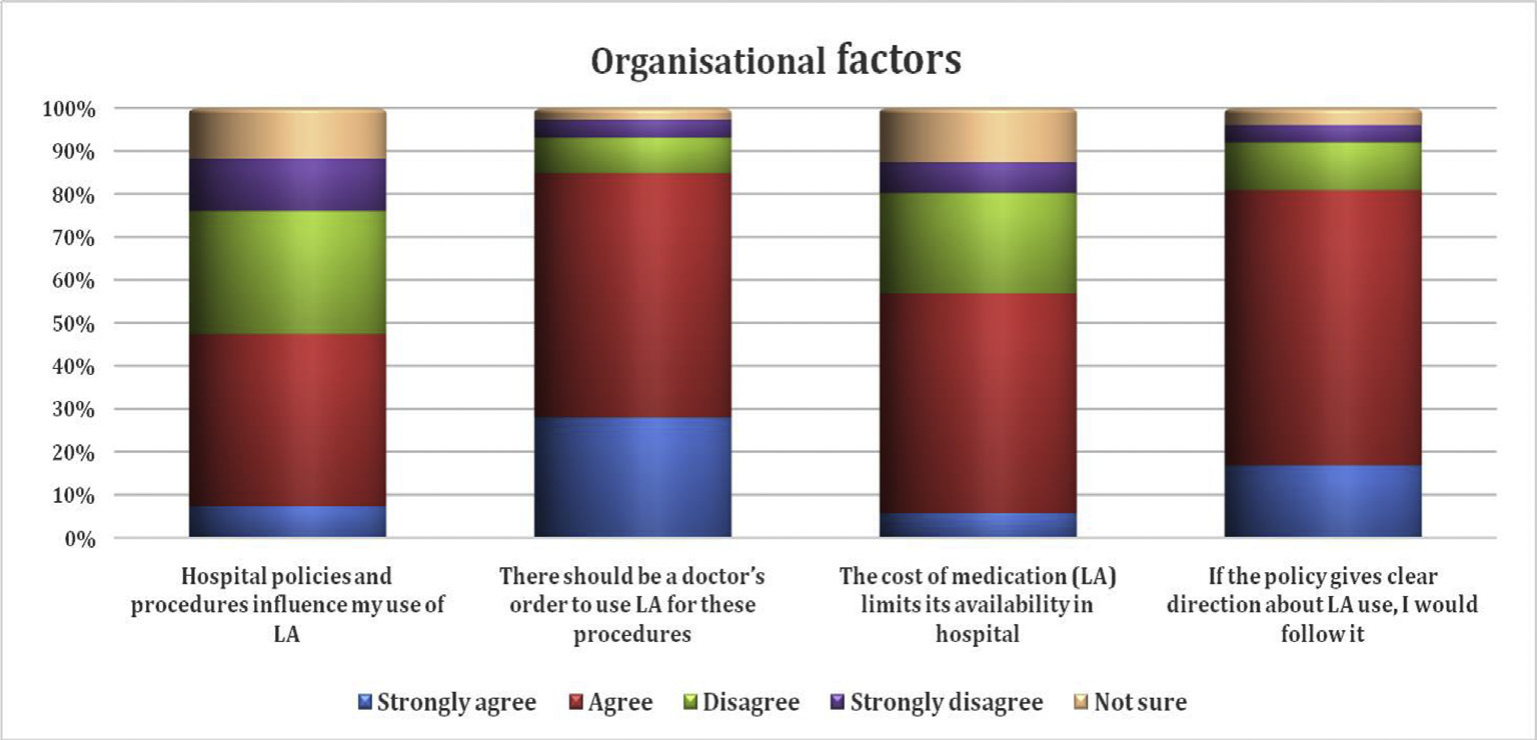
Organisational factors.

Organisational factors that can influence LA use during needle procedures included hospital policy, doctors' orders, and medication costs. Most respondents agreed (i.e., answered agree or strongly agree) that there should be a doctor's order to use LA (84.8%) and that they would follow hospital policy if it gives clear instructions about LA use (80.8%) (Figure 2).

**Figure 3:**
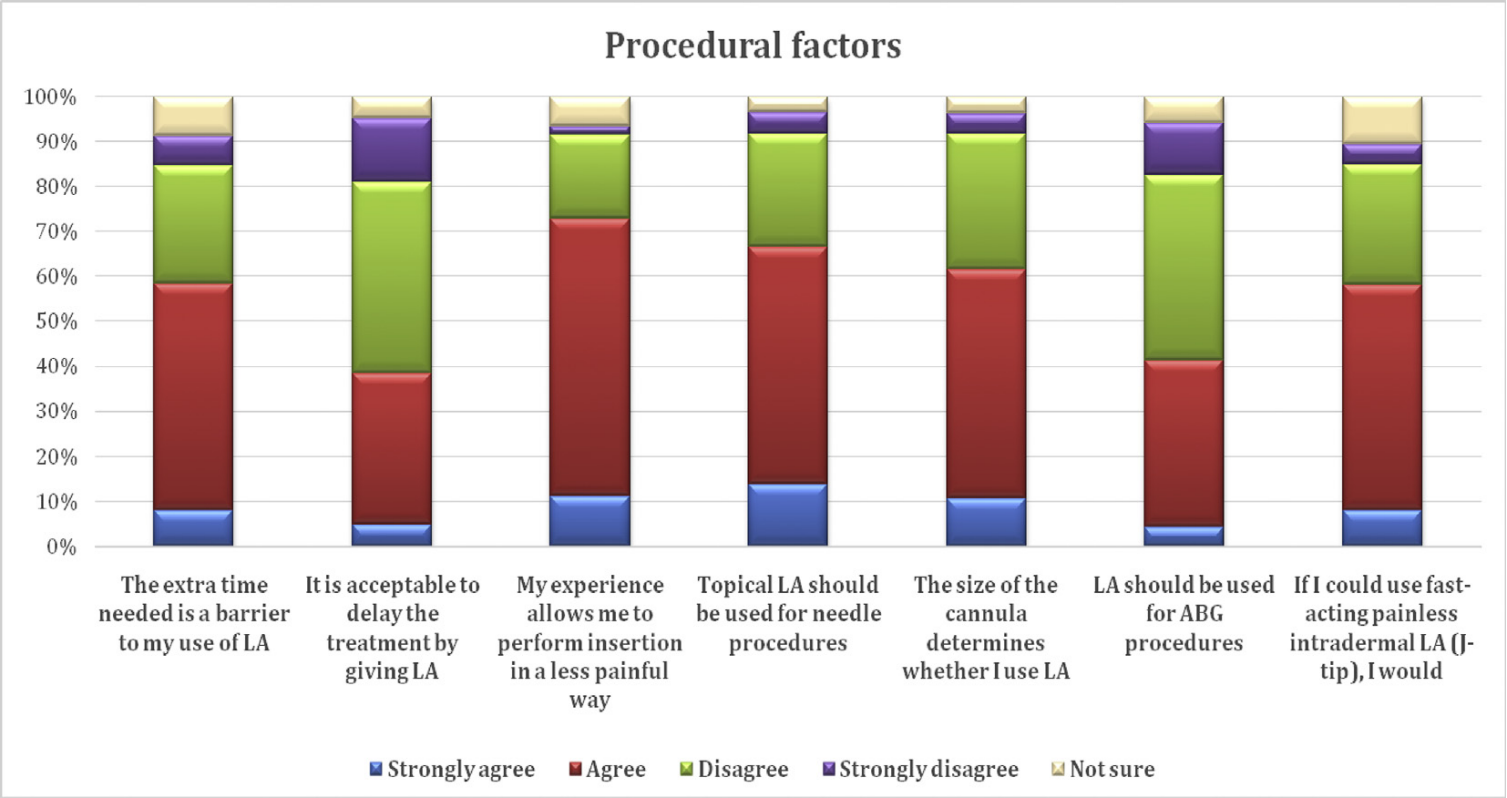
Procedural factors.

The procedural factors related to LA use were time, nurses' experience, LA type (topical or injection), and procedure type (IV cannulation or ABG). Participants predominantly agreed with five out of these seven items, with nurses’ experience having the highest rate of agreement (72.9%), followed by topical LA (66.5%); similar rates were found for extra time, cannula size, and painless intradermal LA (Figure 3).

**Figure 4:**
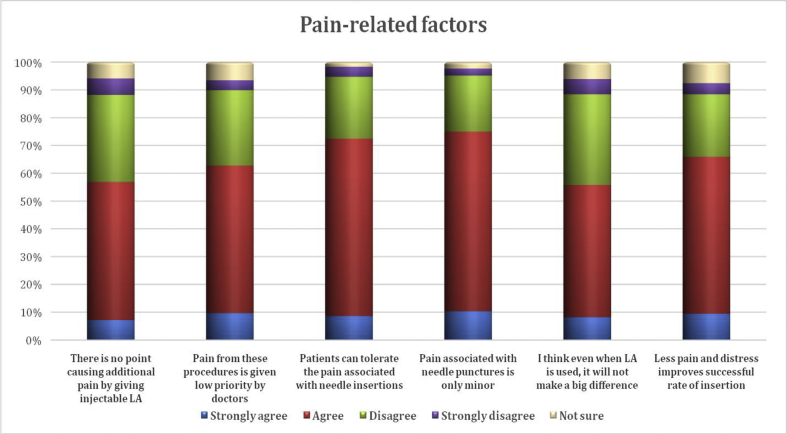
Pain-related factors.

Of the relevant pain-related factors, the belief that needle pain is only minor and that patients can tolerate needle pain were highest (74.9% and 72.4%, respectively). Higher rates of disagreement were found for the items of causing additional pain (37.3%) and that LA use will not make a big difference (38.1%) (Figure 4).

**Figure 5:**
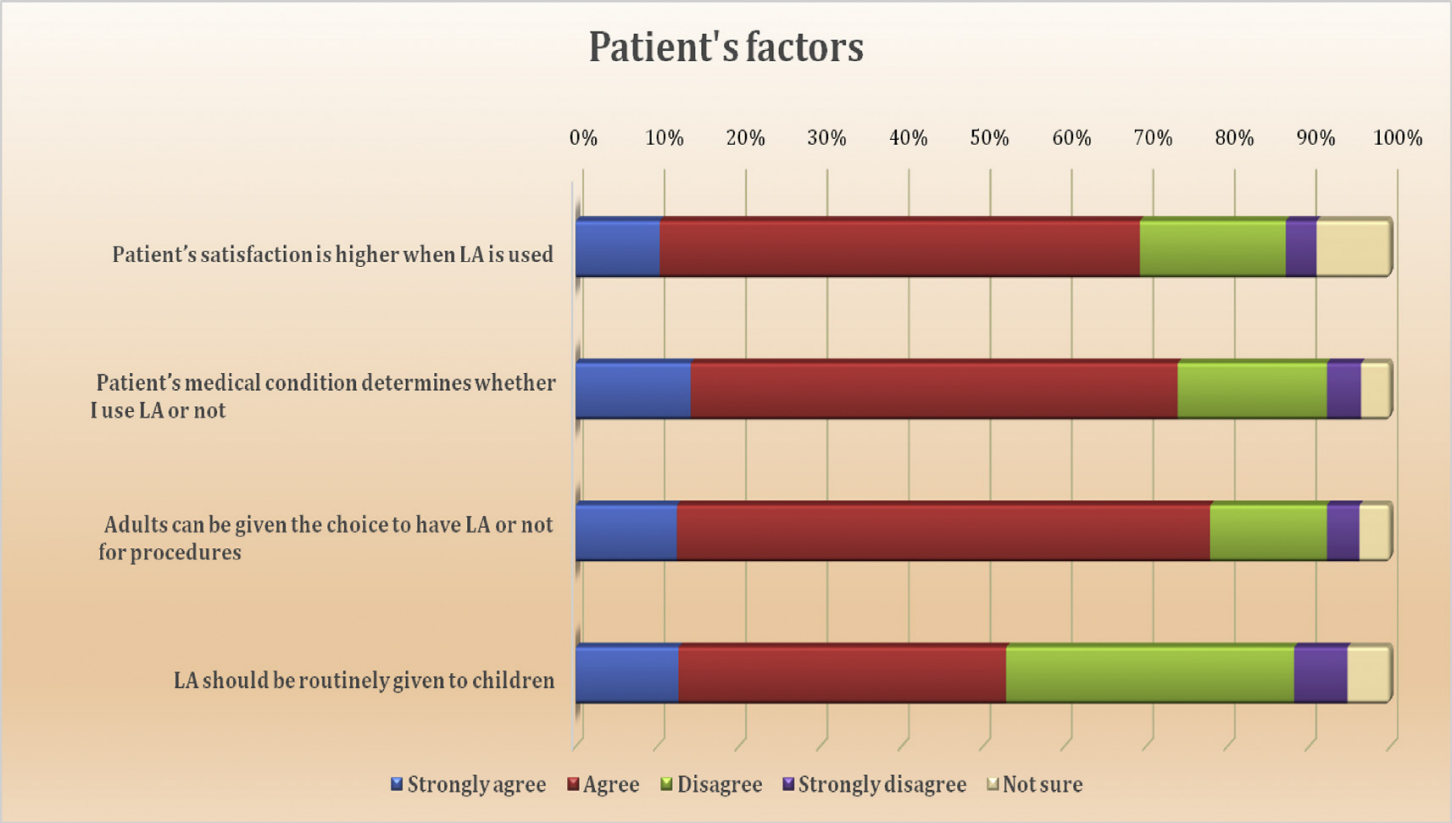
Patient's factors.

Patient-related factors included patient's age, medical conditions, and satisfaction. Overall, the respondents predominantly agreed with all these patient-related factors. The agreement rate for three factors was far higher than the disagreement (adults can be given the choice, 77.9%; the patient's medical condition, 73.9%; and patient satisfaction, 69.2%). However, for the item of giving LA to children, only 53% of participants agreed, while around 42% indicated disagreement (Figure 5).

**Figure 6:**
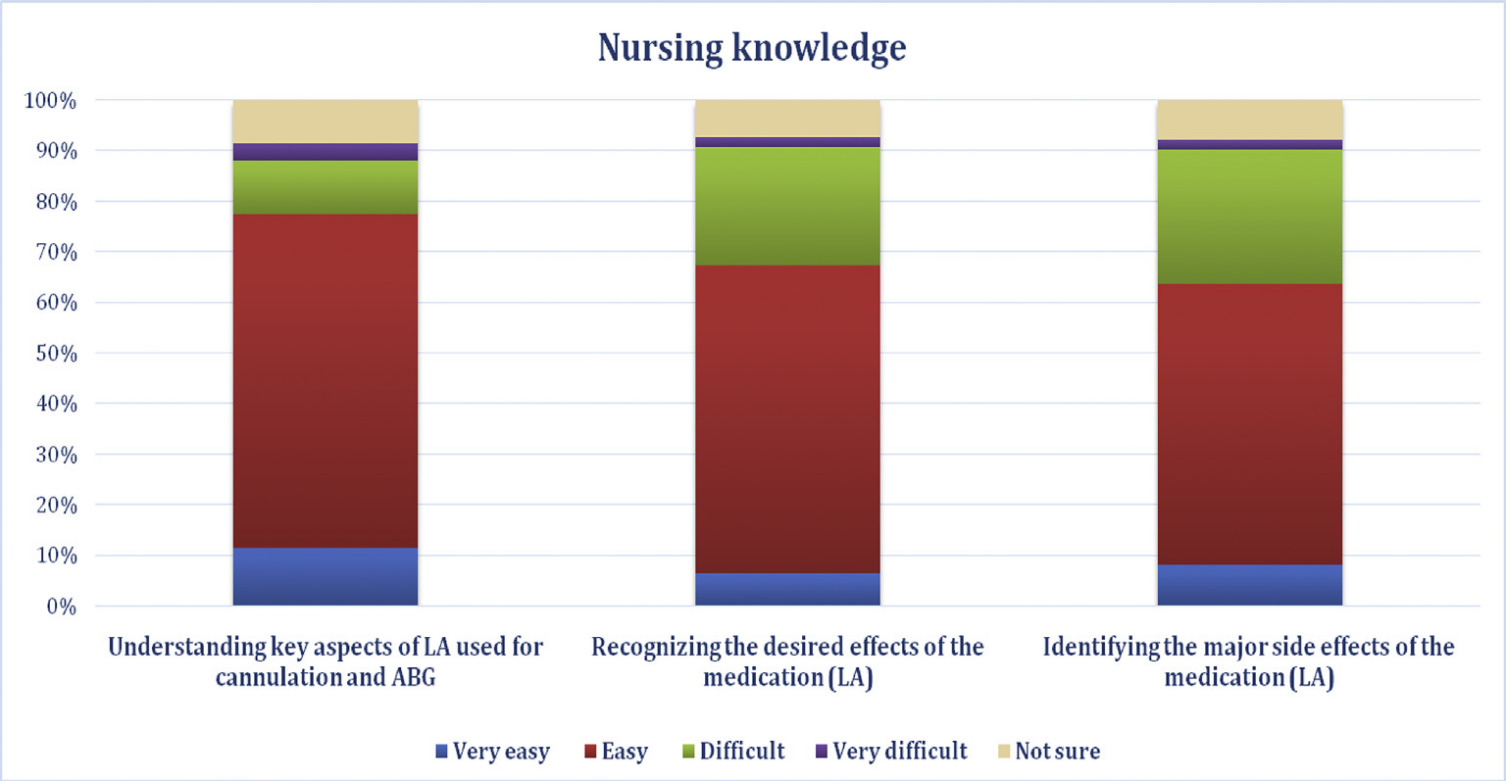
Nursing knowledge.

Nurses’ self-reported knowledge of LA could influence LA use. Most nurses perceived that LA is easy to understand. The item of “understanding the key aspects of LA” had the highest easiness rating (77.5%), with only 31% of participants finding it difficult (Figure 6).

**Figure 7:**
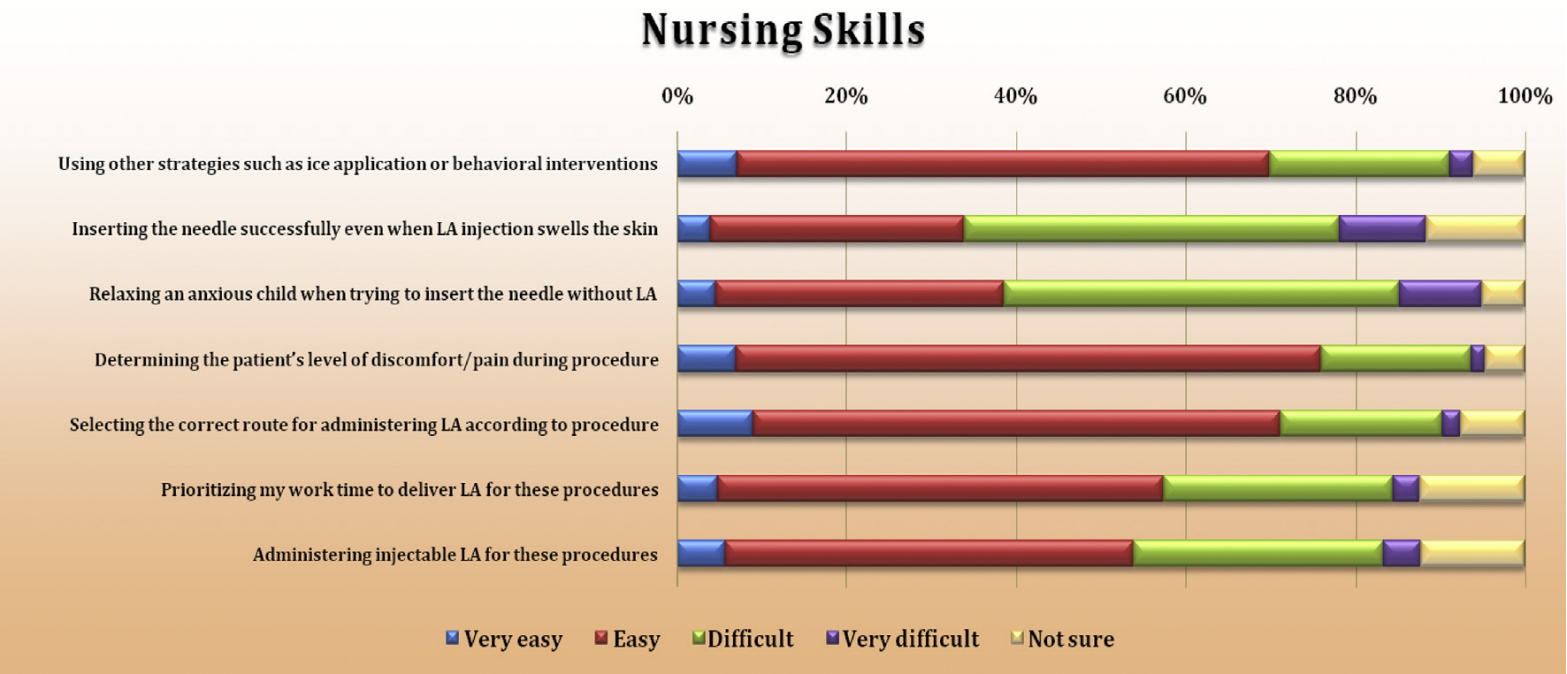
Nursing skills.

Nurses' self-rated skills regarding needle insertions and LA use could also influence LA use. Patient’ level of pain was rated as easiest, with a total of 76% compared to 19.4%. Selecting the correct route for administering LA and using other strategies had similar rates (around 70% considered these easy, and a third considered them difficult). Relaxing an anxious child when inserting the needle was perceived as the most difficult task, with 56.4% of staff giving this response (Figure 7).

### 3.2 Path analysis

**Figure 8:**
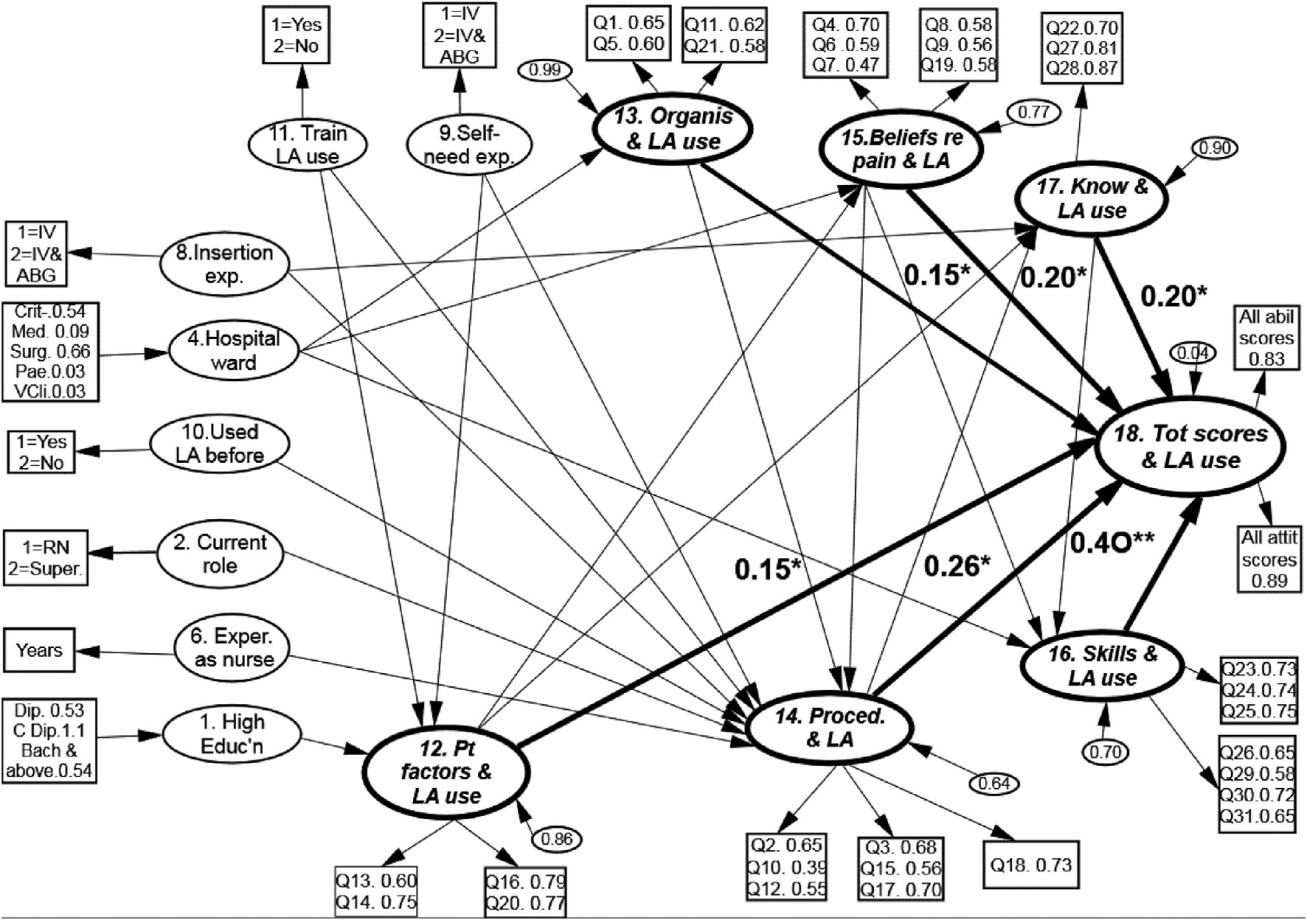
The final model predicting the variables that influence the use of local anaesthetics (∗*p* = 0.05 and ∗∗*p* = 0.001). The factors that directly influenced local anaesthetic use are identified by the six (bold) arrows that point to the local anaesthetic use (total score variable no. 18), and the magnitude of that variable's influence is indicated by the coefficient number next to each arrow. The higher the coefficient value the stronger the relationship is between the two inter-connecting variables (Blackman et al., 2015). Statistically significant variables that exerted indirect influences are represented using non-bolded arrows. IV = intravenous; ABG = arterial blood gas; LA = local anaesthetic; exp. = experience; Exper = experience; Organis = organisation; know. = knowledge; Tot = total; Proced. = procedure; Pt = patient; Educ'n = education; Crit = critical settings; Med. = medical; Surg. = surgery; Pae. = paediatrics; VCli. = various clinics; RN = registered nurse; Super = supervisor; Dip = diploma; Coll Dip = college diploma; Bach = Bachelor's degree.

Figure 8 identifies six significant variables directly influencing LA non-use for needle procedures. In addition, there were interrelationships between these six factors.

The LV ‘clinical skills’ (LV 16) had the greatest influence on LA use for needle procedures (IV cannulation and ABG) (path coefficient 0.40). This finding suggests that nurses who perceive that LA use is easy are more likely to use it. Procedural factors (LV 14), such as time concerns, insertion experience, LA type, and procedure type (IV or ABG), had a significant and direct influence on LA use (path coefficient 0.26). The staff's knowledge about LA (no. 17) and their ability to apply it during parenteral procedures had a direct and significant effect on LA use (0.20). Pain-related factors (LV 15), such as underestimation of needle pain, also significantly influenced LA use (0.20), as did organisational factors (LV 13), such as estimating staff's beliefs about the importance of hospital policy and LA use (0.15). Patient demographics, such as their age and medical condition (LV 12), directly influenced LA use (0.15). Furthermore, eight variables exerted indirect influences (Figure 8 and Table 3), which we explore below in detail.

**Table 3:** Descriptions of the factors accounting for the indirect effects on local anaesthetic use.

Number and name of the variable being influenced in turn by another factor (exerting an indirect effect on the final LA use scores [LV 18])	Factor exerting an indirect influence on the direct variable	Magnitude of the variables' indirect effects on all LA use scores (LV 18)
12. Patient factors	1. Highest qualification	0.13
	9. Self-needling experience	0.13
	11. Trained to use LA	0.13
	13. Organisational factors	0.47
13. Organisational factors	4. Hospital ward type	-0.17
	9. Self-needling experience	-0.73
14. Procedural factors	6. Years of nursing experience	-0.92
	8. Insertion experience	0.70
	10. Used LA before 2. Current work role	-0.65
	2. Current work role	0.60
	15. Beliefs about pain and LA	0.32
	13. Organisational factors	0.23
	11. Trained to use LA	0.07
	9. Self-needling experience	-0.05
	12. Patient factors	0.37
15. Beliefs about pain and LA	12. Patient factors	0.30
	4. Hospital ward type	-0.10
	13. Organisational factors	0.43
16. Skills in LA use	17. Knowledge about LA	0.58
	15. Beliefs about pain and LA	0.15
	4. Hospital ward type	-0.07
17. Knowledge about LA	12. Patient factors	0.30
	14. Procedural factors	0.30
	8. Insertion experience	0.07

LA = local anaesthetic; LV = latent variable.

#### 3.2.1 Nurses’ self-efficacy regarding their clinical skills for using LA (LV 16)

The influence of nurses’ knowledge (LV 17) on their skills was significant and strong (0.58) and it confirmed that staff who believe themselves more capable of applying their nursing knowledge feel more confident about their clinical skills for LA use. Furthermore, nurses agreed that pain-related factors (LV 15) are important issues associated with LA use for needle procedures, which influenced their beliefs about their own needle-procedure skills (LV 16; 0.15). The relationship between the hospital wards and nursing skill variables (-0.07) indicated that nurses working in critical wards agreed more strongly than those in other clinical areas.

#### 3.2.2 Procedural factors that influence LA use (LV 14)

A path coefficient of 0.37 was found between procedural factors (LV 14) and patients’ factors (LV 12), indicating that nurses' beliefs about individual patient factors are important for influencing their decision to use LA as well as their beliefs about procedural factors. Procedural factors were also associated with organisational factors (LV 13) and pain-related factors (LV15) (0.23 and 0.32, respectively).

Nurses' needle-insertion experience (no. 8) had a strong impact on their beliefs about using LA as a procedural component (LV 14; 0.7). Compared to nurses with insertion experience in both IV cannulation and ABG, nurses with only IV cannulation experience agreed that procedural factors and LA use are highly important. The staff's current role (LV 2) significantly influenced their beliefs about procedural factors and LA use (0.6); staff nurses (who represent 90.5% of the population) more strongly agreed with the notion that procedural factors should be considered when using LA than did supervisors/head nurses. Having been taught or trained to use LA (LV 11) had a weak influence on the staff's beliefs about LA use (0.07). This indicates that prior education or training for using LA prior to parenteral procedures is an important factor when considering LA use for needle procedures.

The inverse relationship between self-needling experience (LV 9) and staff's beliefs about using LA for needling procedures (-0.05) confirmed that nurses who had experienced needle insertion themselves for both procedures (IV and ABG) had stronger opinions about LA use than did those who had experienced IV cannulation only. In addition, nurses who had never used LA for parenteral procedures (LV 10) agreed more strongly with LA use than staff who had actually administered it (-0.65). The negative coefficient of -0.92 for LV 6 (nursing experience) indicated that nurses with less clinical experience agreed with the values associated with using LA compared to the more experienced staff.

#### 3.2.3 Nurses’ knowledge about LA for parenteral procedures (LV 17)

This variable was modified by two other variables: patient factors (LV 12) and procedural factors (LV 14). Both factors had an equivalent influence (0.3) on the nurses' overall knowledge about LA use. The staff's cannula insertion experience (LV 8), exerted a limited but significant (0.07) impact on their parenteral and needling knowledge.

#### 3.2.4 Pain-related factors associated with LA use for parenteral procedures (LV 15)

Clearly, the staff's beliefs about parenteral pain and LA use were important to them but their beliefs about patient parenteral pain were modified by two other intervening variables. These included the organisation's role (LV 13) and the individual patient factors (LV 12), with coefficients of 0.43 and 0.3, respectively. Additionally, the clinical area (hospital ward; LV 4) influenced the nurses' beliefs about parenterally derived pain and LA use with a coefficient of -0.1, with nurses working in medical, surgical, and other areas (non-clinical) underestimating procedural pain more than staff working in critical and paediatric wards.

#### 3.2.5 Organisational factors that influence LA use (LV 13)

The relationship between the staff's self-needle experience and organisational factors was strong (0.73). This suggests that nurses who have experienced IV cannulation agreed more strongly that organisational factors are important and relevant factors to consider when using LA. The hospital ward type (LV 4) also influenced the staff's beliefs that organisational factors affect their decision to use LA (coefficient -0.17). The path model suggested that it is the nurses working in critical settings who disagree that the doctor's orders and cost of LA are relevant factors to consider regarding using LA.

#### 3.2.6 Patient factors that influence LA use (LV 12)

With a coefficient of 0.47, the staff's beliefs that organisational factors (LV 13) strongly influenced their beliefs about LA use were related to the remaining three demographic variables: education level (LV 1), prior LA training (LV 11), and self-needling experience (no. 9), with each having an identical coefficient of 0.13. Thus, staff who had higher qualifications, had trained in LA, and had experienced needling themselves had stronger opinions about the individual patient factors important to consider when using LA.

## 4 Discussion

### 4.1 The need for a specific LA policy for parenteral procedures

There was a high level of agreement among participants that they should adhere to the hospital's policy as it provides a clear direction for LA use for parenteral procedures. This result showed that nurses follow the hospital's policy as a legal requirement in their profession. The main issue in this study is that there is no hospital policy directing LA use for parenteral procedures. The path model also demonstrated that there is a large variation amongst the staff's beliefs as to whether LA use is acceptable or not. With such variation in beliefs and the subsequent impact on professional nursing behaviour, there is a strong need for a consistent and transparent parenteral LA policy that is based on evidence.

### 4.2 The need for doctors’ orders to use LA for parenteral procedures

Most participants (about 85%) believed that doctors' orders to use LA is a factor that can facilitate its use for parenteral procedures. This finding is consistent with past literature showing that doctors' non-authorisation to use LA before parenteral procedures is a barrier to nurses’ ability to provide optimal pain management (Czarnecki et al., 2011; Hudson et al., 2006; Papa and Zempsky, 2010). Furthermore, the path analysis showed that over 60% of nurses believed that doctors underestimate pain from IV cannulation and ABG procedures, which influenced their attitudes towards LA use. This is evident in this study as most nurses underestimated needle pain and believed that LA will not make a big difference in reducing pain.

From the path model, it seemed that nurses’ clinical judgements and decisions are influenced by administrative policy and medical decisions. In other words, if using LA is good for patients, then this would dominate hospital policy or doctors would order it. Thus, in the absence of an appropriate hospital policy and of medical orders to prescribe LA, current nursing practice is shaped by these limitations or barriers, and LA is not used for needling procedures.

### 4.3 The cost of LA is a factor influencing its availability in hospitals

This study's findings showed that most participants viewed the cost of LA as a factor influencing its availability in the hospital. This finding is consistent with research that identified that one reason for not using topical LA (EMLA cream) (prior to parenteral procedures) was that it was very costly (Burke et al., 2011). Therefore, this study considers that the cost of LA is important when summarising choices for its use in parenteral procedures because it can indeed be prohibitive to its use.

### 4.4 Time constraint as a barrier to LA use

This study demonstrated that, irrespective of what clinical area the nurses worked in, they had time concerns about administering LA prior to parenteral procedures that added to their workload and/or delayed the treatment. These concerns are supported by another earlier study (Czarnecki et al., 2011). Nurses' resistance to new practices is related to the influence of professional socialisation on the nursing practice while being portrayed as being ritualistic and non-patient-focused (Mooney, 2007). Additionally, the nurses' resistance to change could relate to the concern that extra demands may add to their workload, which may restrict their time to fulfil these demands. This practice reflects a task-focused approach that uses the time factor as an excuse instead of examining what is best for patients when providing ultimate care. These issues need to be addressed during hospital staff development and undergraduate nursing education to increase nurses’ knowledge of developing assertion, reflection, and critical thinking skills and providing patient-centred holistic care.

### 4.5 Nurses’ beliefs in performing needle insertions painlessly

The results showed that over 70% of the respondents believed that their experience in performing needle procedures allowed them to perform needle insertion in a less painful way. This indicates that experienced nurses' belief in their ability to insert the needle painlessly negates the need for using LA in parenteral procedures. This assumption is false because there are many factors that can affect the degree of pain arising from parenteral procedures (apart from the length of experience) including the patient's age, procedure type (arterial or venous access), difficulty with their blood vessels, and needle size. Furthermore, pain is subjective and only the patient can determine its intensity.

### 4.6 LA type as a determinant of using it for parenteral procedures

This study found near total agreement on the use of a painless type of LA (topical and Intradermal jet injector (needle-free) called J-Tip device), but less agreement on giving LA for ABG procedures and minimal agreement on giving LA to children on a routine basis. One reason for these mixed responses could be that LA is commonly thought of as an injection, while LA use can in fact take different forms (injection, spray, patch, or ointment). The nurses had a predominant belief that giving LA causes additional pain, and nurses are more concerned about a needle being inserted twice (LA injection and procedure needle). This outcome shows that nurses lack knowledge about the LA's effect and the alternatives to injectable LA for numbing the skin (insertion site) that eliminate the secondary needle puncture pain (Burke et al., 2011; Matheson et al., 2014; McNaughton et al., 2009). This lack of knowledge is a barrier to providing painless parenteral procedures for patients, and nurses should be educated about the effect of LA to enhance their knowledge and change their attitudes towards accepting its use for patients. In addition, different LA types should be provided in the hospital to suit both the patients' and nurses' preferences.

### 4.7 Nurses’ experiences with parenteral procedures

The path model showed that nurses who have personally experienced both IV and ABG procedures agree more that the procedural factors (e.g. time concerns) are more prominent issues when considering LA use. This finding is different than anticipated as this group was more sensitive to the consideration of using LA because of their personal experience of the procedural pain. Especially, the ABG procedure is more painful than IV access because it is more invasive due to the artery's anatomical structure (Hajiseyedjavady et al., 2012; Hudson et al., 2006). This finding also contradicts that of McNaughton et al. (2009) in which self-needling experience influenced health professionals' attitudes towards using LA after their experience of needle pain with and without LA. Thus, the participants' views were based only on their needle-pain experience without having used LA to perceive the difference.

### 4.8 Nurses' beliefs about patients’ pain experiences (arising from needling procedures) and LA use

One major factor that caused nurses to not consider using LA for parenteral procedures was their underestimation of the pain associated with needle punctures. This finding is consistent with other studies (Hudson et al., 2006; Sado and Deakin, 2005). This perception is problematic. First, pain is a subjective experience that relies on the patient's report. Second, patients' tolerance for pain varies considerably, and nurses' estimation of the patients' levels of pain can be inaccurate (Olsen, 2016). Thus, nurses are being unreliably judgemental and making generalisations about the degree of discomfort patients experience rather than relying on the patients' reported or lived experience (Olsen, 2016). Additionally, lack of knowledge is evident from the study's results, as most nurses agreed that using LA will not make a big difference in reducing procedural pain. One reason for this could be that most respondents (just over 80%) had never used LA before and had not been taught or trained to use it.

Interestingly, however, despite the nurses' reluctance to use LA and their underestimation of needle pricks, the majority agreed that patient satisfaction is higher when LA is used. In addition, most nurses thought that less pain and distress would improve successful insertion rates. These conflicting views suggest that nurses who acknowledge the benefit and comfort that LA can provide still believe that they cannot change current practice because of other factors and issues, such as organisational requirements. Generally speaking, it seems that nurses' clinical judgement is influenced more by organisational decisions, and does not consider the patients' best interests, which leads to failure in reference to the core obligation of nursing to alleviate pain, advocate for the patient's benefit, and respect their autonomy.

### 4.9 Patients’ demographic factors as determinants of LA use for parenteral procedures

There was overall agreement that the patient's demographic factors, including their age and medical condition, are important factors when considering using LA. The staff differentiated who needs to have LA (prior to needling procedures) according to their age. Just 52.8% agreed that LA should be given to children routinely before parenteral procedures, while giving LA to adults as a choice achieved greater consensus (78%). This issue of patient choice is interesting in that, firstly, it was not an underlying assumption for children's pain management and, secondly, it suggests that nurses believe that adults can tolerate pain (more than children) and thus do not need LA routinely. Furthermore, the path model showed a link between this factor (patient's medical condition) and a treatment delay factor. In Papa and Zempsky (2010), nurses working in emergency departments expressed their reluctance to provide LA for procedural pain because patients with critical conditions require rapid responses and administering LA may delay treatment. Additionally, the current study demonstrates that nurses share this view.

### 4.10 Nurses’ knowledge and skills with LA and parenteral procedures (self-efficacy)

Self-efficacy is an individual's belief in their ability to perform a task (Bandura, 1986; Shortridge-Bagget, 2002). This study's path model showed a very strong relationship between the nurses' LA knowledge and their self-efficacy estimates about implementing LA. In other words, their knowledge and agreement levels about different aspects of LA use was a strong predictor for their perception of being able to both understand and administer LA. High levels of self-efficacy do not necessarily mean that the nurse can actually perform this clinical skill (i.e. administer LA) competently; however, their motivation to engage with this skill and readiness to learn how to administer LA prior to needling procedures can now be predicted.

## 5 Limitations

This study's findings are only valid in the context of its sample, which was nurses working in one hospital in Saudi Arabia. Additionally, the study did not measure self-efficacy for specific knowledge about LA, which may have caused variation in the responses. Further study is needed to investigate these reported factors from the patients' perspectives and to compare them to the nurses' perspectives.

## 6 Conclusion

The nursing staff's capacities to utilise LA for parenteral procedures were modelled and predicted. The significant predictors that directly influenced the staff's attitudes and self-efficacy scores included organisational factors, such as hospital policy and the doctor's orders, which were major factors influencing the preference for using LA for needle procedures. However, it is noted that nursing practices were shaped by the following procedural factors. Time constraints were identified as a barrier that affects the decision to use LA for needle insertions because LA administration may increase the nurses' workload and delay treatment. Additionally, underestimation of needle pain was a major factor that was influenced by the abovementioned organisational factors. Finally, the patients' individual factors, such as age and medical conditions, influenced nurses' attitudes regarding using LA for needle insertions.

## Declarations

### Author contribution statement

Fatimah Alobayli: Conceived and designed the experiments; Performed the experiments; Analyzed and interpreted the data; Contributed reagents, materials, analysis tools or data; Wrote the paper.

Ian Blackman: Analyzed and interpreted the data; Contributed reagents, materials, analysis tools or data.

### Funding statement

This research did not receive any specific grant from funding agencies in the public, commercial, or not-for-profit sectors.

### Competing interest statement

The authors declare no conflict of interest.

### Additional information

No additional information is available for this paper.
