# Corrigendum to “Modelling nurses” use of local anaesthesia for intravenous cannulation and arterial blood gas sampling: A cross-sectional study” [Heliyon 6 (3) (2020) e03428]

**DOI:** 10.1016/j.heliyon.2020.e03605

**Published:** 2020-03-30

**Authors:** Fatimah Yahya Alobayli, Ian Blackman

**Affiliations:** a King Saud Medical City, Ulaishah, Riyadh, 12746, Saudi Arabia; b Flinders University, School of Nursing & Midwifery, Sturt East Buildings, Bedford Park, SA, 5042, Australia

In the original published version of this article, the text did not cite the following source as a reference: Alobayli, F.Y., 2019. Factors influencing nurses' use of local anesthetics for venous and arterial access. J. Infusion Nurs. 42 (2), 91. The authors apologize for this error. Both the HTML and PDF versions of the article have been updated to correct the error.

